# Identification of small molecules capable of enhancing viral membrane fusion

**DOI:** 10.1186/s12985-023-02068-1

**Published:** 2023-05-24

**Authors:** Mª Jesús García-Murria, Laura Gadea-Salom, Sandra Moreno, Marina Rius-Salvador, Oscar Zaragoza, Alejandro Brun, Ismael Mingarro, Luis Martínez-Gil

**Affiliations:** 1 Departament de Bioquímica i Biologia Molecular, Institut Universitari de Biotecnologia i Biomedicina (BIOTECMED), Universitat de València, Burjassot, E-46100 Spain; 2 Centro de Investigación en Sanidad Animal, CISA (Instituto Nacional de Investigación y Tecnología Agraria y Alimentaria/Consejo Superior de Investigaciones Científicas (INIA/CSIC)), Madrid, Spain; 3 Mycology Reference Laboratory, National Center for Microbiology, Instituto de Salud Carlos III, Majadahonda, Madrid, Spain; 4 Center for Biomedical Research in Network in Infectious Diseases (CIBERINFEC, Health Institute Carlos III, CB21/13/00105), Madrid, Spain

**Keywords:** SARS-CoV-2, Influenza a virus, Nipah virus, Enveloped virus, Viral entry, Membrane fusion, Syncytium, HTS, High-throughput screening.

## Abstract

Several approaches have been developed to analyze the entry of highly pathogenic viruses. In this study, we report the implementation of a Bimolecular Multicellular Complementation (BiMuC) assay to safely and efficiently monitor SARS-CoV-2 S-mediated membrane fusion without the need for microscopy-based equipment. Using BiMuC, we screened a library of approved drugs and identified compounds that enhance S protein-mediated cell-cell membrane fusion. Among them, ethynylestradiol promotes the growth of SARS-CoV-2 and Influenza A virus in vitro. Our findings demonstrate the potential of BiMuC for identifying small molecules that modulate the life cycle of enveloped viruses, including SARS-CoV-2.

**Supplementary Information:**

The online version contains supplementary material available at 10.1186/s12985-023-02068-1.

## Introduction

The novel severe acute respiratory syndrome coronavirus-2 (SARS-CoV-2) [1, 2] has caused the pandemic disease known as coronavirus disease 2019 (COVID-19). This disease has had and continues to have, a tremendous impact on our healthcare systems and the global economy. Accordingly, extraordinary efforts have been made to develop prophylactic and therapeutic measures to reduce the morbidity and mortality associated with COVID-19 disease.

The cellular membrane is the first barrier the virus encounters when infecting a new cell. At some point during entry, enveloped viruses must fuse the cellular and viral membranes. In the case of SARS-CoV-2, the highly glycosylated spike (S) protein is responsible for gaining entry to the host cells [3]. The S protein, a class I trimeric fusion protein [4], is translated in a non-active form (S_0_). Proteolytic activation of S_0_ by host proteases (i.e. TMPRSS2) produces the mature pre-fusion S protein incorporated in the virions [5]. The infectious process starts with the binding of S protein to the angiotensin-converting enzyme 2 (ACE2) at the cell surface [2, 6]. Binding to ACE2 triggers a conformational change, resulting in the exposure of the S protein fusion peptide, which will interact with the host membrane and initiate the fusion of the viral and cellular membranes. Facilitating any of the multiple steps in this complex process would speed up and increase viral production, expediting traditional vaccine development, reducing production costs, and increasing manufacturing yield [7].

Multiple approaches have been developed to safely analyze viral entry, including pseudo-typed virus-based assays, viral-like particles-based assays, biochemical assays, or cell-cell fusion assays. Syncytium identification, because of the expression of the viral fusion machinery and the corresponding host receptor on the cellular surface, has traditionally been done by microscopy. However, microscope-based methodologies hinder the quantification of the fusion process and the implementation of high throughput methods for small molecule identification.

To study the SARS-CoV-2 S-mediated membrane fusion process, we decided to adapt the recently developed Bimolecular Multicellular Complementation (BiMuC) assay [8]. Based on the bimolecular complementation properties of a fluorescent reporter protein, this assay facilitates the identification and quantification of viral-induced cell-cell fusion events without the assistance of microscope-based equipment. After accommodating BiMuC for the study of SARS-CoV-2, we set up a screen for small molecules capable of modulating the S protein-mediated membrane fusion process. We screened 1280 small molecules, using this assay, and identified that ethynylestradiol enhances S protein-mediated cell-cell membrane fusion. As a result, ethynylestradiol augments the growth of SARS-CoV-2 in vitro. Furthermore, the effect on viral-mediated membrane fusion is not restricted to SARS-CoV-2. We demonstrated that ethynylestradiol could increase Influenza A virus growth and Nipah virus membrane fusion. Our results show that BiMuC could be implemented to monitor the SARS-CoV-2 membrane fusion process and identify small molecules that perturb this process.

## Materials and methods

### Cell lines and plasmids

Hek 293T, Vero E6, and Madin-Darby canine kidney (MDCK) cells were obtained from ATCC (http://www.atcc.org) and were maintained in Dulbecco’s Modified Eagle Medium (DMEM) (Gibco, http://www.lifetechnologies.com) supplemented with 10% fetal bovine serum (FBS, Gibco). The SARS-CoV-2 S plasmid was a gift from Dr. F. Krammer (Ichan School of Medicine at Mount Sinai). The Jun-Nt VFP (Jun) and Fos-Ct VFP (Fos) and the human ACE2 and TMPRSS2 expressing plasmids were obtained from Addgene (#22,012, #22,013, #1786, and #53,887, respectively).

### BiMuC assay

To study SARS-CoV-2 membrane fusion properties, Hek 293T cells (DMEM supplemented with 10% FBS) were transfected using polyethyleneimine (PEI) [9], either with SARS-CoV-2 S and Jun or with ACE2 and Fos plus the pRL Renilla Luciferase (Promega) to normalize the signal. Additionally, we tested the SARS-CoV-2 S-Fos and the ACE2-Jun combinations, once again, with the pRL Renilla Luciferase. The next day cells were counted, mixed, and seeded into 96 well plates (1 × 10^5^ cells/well in 100 µL of media). After 48 h, fluorescence was measured (λ_exc_ = 485 nm, λ_em_ = 535 nm) on 96 well plates as previously described [10].

For the identification of small molecules with the ability to modulate membrane fusion, the protocol was adjusted to meet the necessary screen standards. To evaluate the robustness of our assay, we calculated the Z′-factor and the Signal-to-Noise (S/N) ratio. *Z*′-*factor* = 1 − ((3δ*pos* + 3δ*neg*)/(µ*pos* − µ*neg*)), where µpos is the mean signal for the positive control, µneg is the mean signal for the negative control, δpos is the standard deviation of the positive control, and δneg is the standard deviation for the negative control. Briefly, Hek 293T cells were seeded in 15 cm dishes (5 × 10^6^ cells/dish) using DMEM supplemented with 10% FBS. After 24 h of incubation (37 °C, 5% CO_2_), cells were transfected using PEI with the plasmid as mentioned above combinations or mock transfected. On the next day, cells were counted, mixed, and manually seeded into solid black 96 well plates (1 × 10^5^ cells/well in 100 µL of media). Cells were mixed into 2 pools, pool A containing cells expressing Jun and SARS-CoV-2 S or Fos and ACE2 and pool B with cells expressing Jun or Fos. Wells in columns 1–11 were seeded with 50 µL cells from pool A, while column 12 received 50 µL of cells from pool B. Next, 50 µL of the small compounds at 50 µM were manually added to the cells (final concentration 25 µM, 0,25% DMSO). We used a Prestwick Chemical library (Sigma, St. Louis, MO, USA) containing 1280 compounds in 96 well plates. Columns 1 and 12 (positive and negative controls, respectively) were treated with DMSO in each plate. After 48 h of incubation, the media was substituted by 100 µL of PBS, and the fluorescence was measured in a VictorX multi-plate reader (Perking Elmer, Waltham, MA, USA). Results obtained from the screen were standardized using the Z-Score, calculated as follows: Z-Score = (x − µ)/δ, where x is the raw signal, µ is the mean signal, and δ is the standard deviation of all the compound-containing wells of one plate. The Z-Score indicates how many standard deviations a particular compound is above or below the mean of the plate. Primary hits were identified by calculating a Z-score for each compound and applying hit selection criteria; Z-Score > 1.5. Hits were confirmed in secondary screening. This time cells included the pRL-Renilla. The luminescence was measured using the Renilla Luciferase Assay kit from Sigma following the manufacturer protocol on 96-well white cell-culture plates. This measure facilitates the elimination of those compounds promoting cell division or survival, thus increasing the fluorescence signal without effectively increasing cell-cell fusion. To discard the hits that had fluorescent properties similar to VFP, cells were seeded in black 96-well plates and treated with the compounds under the same conditions as described above. In this case, fluorescence was measured as before (λ_exc_ = 485 nm, λ_em_ = 535 nm). In all assays, [DMSO] was kept at ≤ 1%. Statistical analysis was done with Libre Office Calc.

To study the effect on the membrane fusion induced by the Nipah virus, we carried out the original BiMuC assay described in [8]. In this case, Hek 293T cells were transfected with Jun-Nt, NiV F, and G or with Fos-Ct and a plasmid encoding Renilla luciferase (pRL, Promega). Next, cell pools were mixed and treated with the indicated compound (DMSO concentration was kept at 1%). After 48 h fluorescence was measured. At least three independent experiments were done.

### Cell viability

The toxicity of the compounds was evaluated with Cell-Titer Glo (Promega) following the manufacturer’s indications. Briefly, cells were seeded (~ 1 × 10^4^ cells/plate) in an opaque white 96-well plate and treated with the appropriate compound at the indicated concentration for 48 h. Next, 100 µl of CellTiter-Glo® reagent was added and let the luminescence signal stabilizes for 10 min. The luminescence was recorded on a VictorX multi-plate reader (Perking Elmer, Waltham, MA, USA).

### Viral infections

Vero E6 cells were infected at a multiplicity of infection (MOI) of 0.01 with SARS-CoV-2. Next, ethaverine, rabeprazole, or ethynylestradiol were added at the appropriate concentration. Additionally, cells were treated with DMSO. After 48 h, supernatants were collected and titered by standard plaque assay in VeroE6 cells. Alternatively, MDCKs cells were infected with IAV/WSN/33 at an MOI of 0.001. Samples were incubated in the absence or presence of ethaverine, rabeprazole, or ethynylestradiol at the indicated concentrations for 48 h. Next, supernatants were collected and titered by the 50% Tissue Culture Infective Dose (TCID_50_) method in MDCK cells. In all assays, [DMSO] was kept at ≤ 1%.

**Fig. 1:**
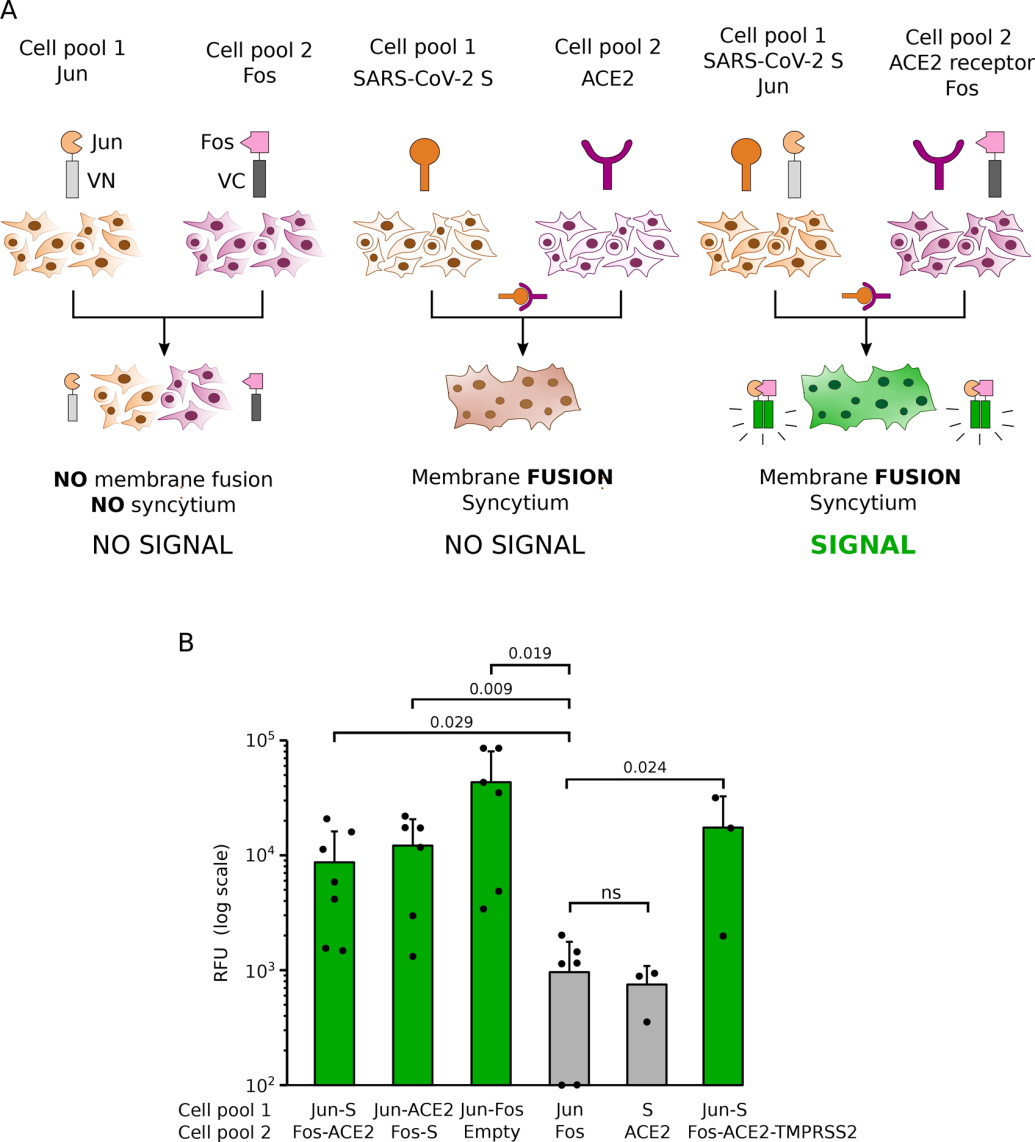
Analysis of SARS-CoV-2 membrane fusion by BiMuC. **A** Schematic representation of the BiMuC assay. The Jun and Fos proteins were fused to the Nt and Ct ends of the VFP, respectively, to create the VN-Jun and VC-Fos chimeras, from now on presented simply as Jun and Fos. Reconstitution of the structure of the VFP (represented in green), and thus its fluorescence properties, occurs exclusively after Jun and Fos interaction. When the VN-Jun and VC-Fos chimeras are expressed in separated cell pools, no reconstitution is possible (left). SARS-CoV-2 membrane fusion relies on the successful interaction between SARS-CoV-2 S and human ACE2. Expressing these two proteins on the surface of two adjacent cells leads to the fusion of the cellular membranes and creating a syncytium (middle). When the cells being fused carry the VN-Jun and VC-Fos chimeras can reconstitute the VFP native structure (depicted in green, left). On the contrary, inhibition of any of the cellular or viral processes that lead to membrane fusion, from synthesis and maturation of the S protein to its interaction with ACE2, will result in the absence of a fluorescent signal. **B** For the cell-cell fusion assay, Hek 293T cells were transfected with the indicated plasmid combinations. That is: Jun + SARS-CoV-2 S, Fos + hACE2, Jun + hACE2, Fos + SARS-CoV-2 S, Jun + Fos, Jun, Fos, SARS-CoV-2 S, hACE2, Fos + hACE2 + TMPRSS2. The next day cells in each cell pool were counted and mixed as indicated. After 48 h, fluorescence was measured. The mean and standard deviation of at least three independent experiments are represented (n values 7, 6, 6, 6, 3, 3, respectively). A solid dot represents the individual value of each experiment. Green bars denote those samples with fluorescence levels comparable to the positive control (Jun-Fos). Statistical differences are based on a two-tailed homoscedastic t-test (p-values are indicated above the corresponding bar, ns non-significant)

## Results and discussion

### Implementation of the BiMuC approach

The novel SARS-CoV-2 is a highly contagious agent. Accordingly, its handling requires substantial safety measures, which may complicate research protocols. To facilitate the study of the SARS-CoV-2 S-mediated membrane fusion process, we decided to implement a fluorescence-based complementation assay [8]. The BiMuC approach is a safe, virus-free approach based on the structural properties of some fluorescent proteins, such as the Venus Fluorescent Protein (VFP) [10–13]. Briefly, the VFP can be split into two fragments (VN and VC, respectively), none of which is fluorescent by itself. However, if these two fragments are fused to a pair of interacting proteins that bring them together, the native structure of the VFP is reconstituted, and its fluorescence properties are recovered. The chimeras are expressed in separated cell pools together with the viral machinery required for membrane fusion. If membrane fusion brings the fluorescent chimeras nearby, the fluorescence would be reconstituted. On the contrary, no signal would be retrieved if the conditions for forming a syncytium were not met (Fig. 1A).

**Fig. 2:**
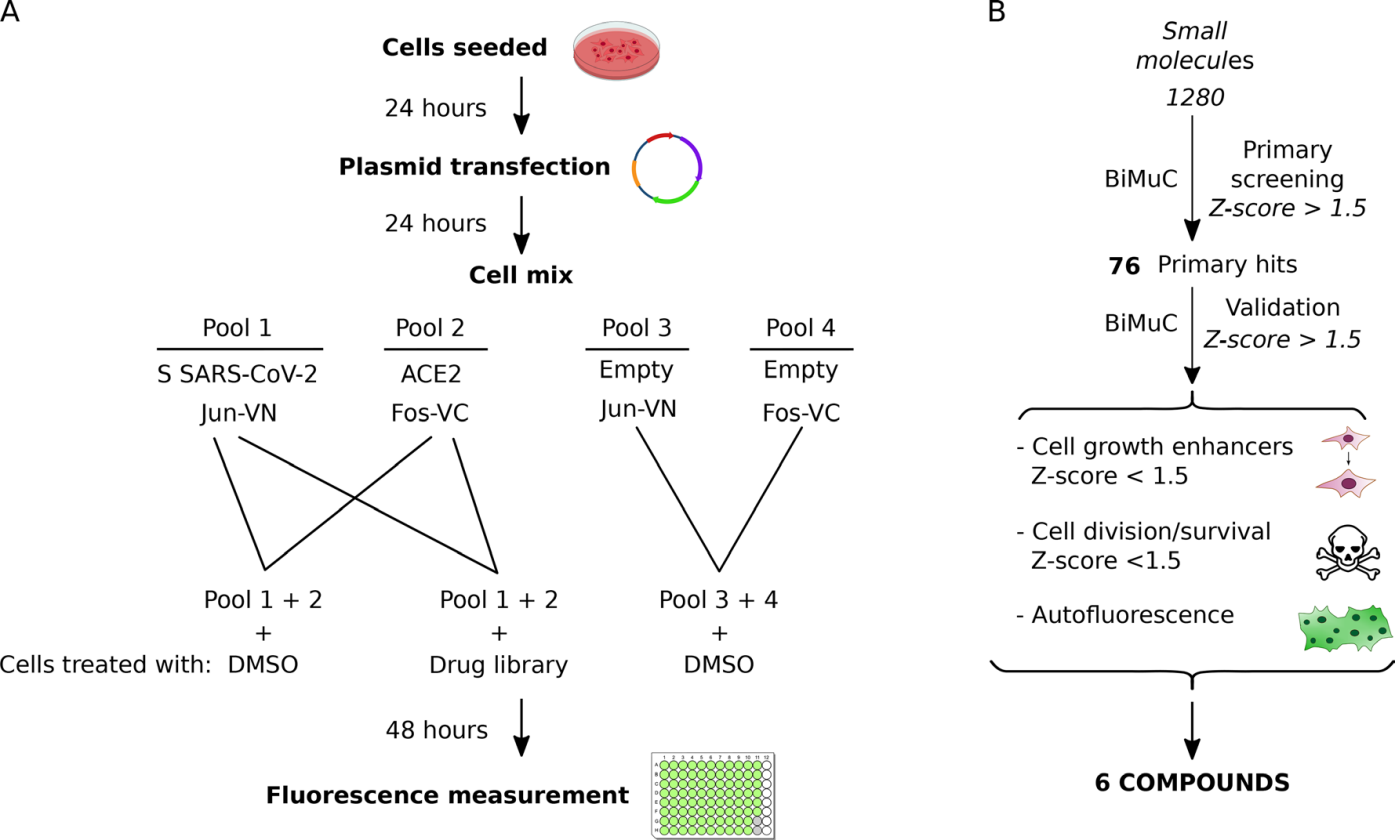
Small molecule identification. **A** Schematic representation of the BiMuC assay used for small molecule identification with membrane fusion enhancing properties. **B** Workflow representation of the small molecule screen and validation of the resulting hits

To test whether we could apply this methodology to the study of SARS-CoV-2-induced membrane fusion, the interacting partners c-Jun and c-Fos [14] were linked to the VN and VC fragments of the VFP, generating the c-Jun/VN and c-Fos/VC chimeras, from now on presented simply as Jun and Fos. These chimeras were expressed in two independent cell pools with SARS-CoV-2 S (Jun-S) and ACE2 (Fos-ACE2), respectively. Additionally, we included cells transfected with Jun and Fos together (Jun-Fos), Jun, Fos, S, ACE2, or an empty plasmid (Empty) in the assay. After transfection, cell pools were combined as indicated and incubated for additional 48 h (Fig. 1B). The samples containing cells transfected with Jun-S and Fos-ACE2 showed a fluorescence signal significantly stronger than those that combined pools of cells expressing Jun and Fos or S and ACE2 (negative controls) suggesting that a successful fusion of the cell membranes triggered by the viral machinery brought the two chimeras together and facilitated reconstitution of the fluorescent signal (Fig. 1B). Note that we also tested the Jun-ACE2 and Fos-S combinations. Our data suggest that the BiMuC methodology could be implemented to safely study SARS-CoV-2 mediated membrane fusion. Images of SARS-CoV-2 S-induced syncytia can be found in Supplementary Fig. 1.

### Small molecule identification

Identifying small molecules that influence the viral life cycle often requires scalable and robust high-throughput screening (HTS) protocols. These procedures should also be safe in the case of highly pathogenic viruses, such as SARS-CoV-2. To demonstrate that the SARS-CoV-2 BiMuC assay could be used in high-throughput conditions, we set up an assay to identify small molecules that influence the SARS-CoV-2 S-mediated membrane fusion process. We were particularly interested in small molecules that could enhance the viral entry. Despite introducing new vaccine platforms, vaccine production often relies on producing large quantities of virus. Accordingly, various strategies have been implemented to increase viral production, from cell selection to optimizing replication procedures [15–17]. A molecule capable of facilitating viral-membrane fusion would, theoretically, enhance viral growth and could be used to facilitate traditional vaccine production [7].

Our assay is based on Hek 293T cells, which express low levels of ACE2 and TMPRSS2 [18, 19]. While ACE2 was supplemented *in trans*, we intentionally did not include TMPRSS2 in our assay to set up a sub-optimal fusion state that facilitates the identification of small molecules that enhance syncytia formation. Speeding up or increasing virus production would expedite vaccine development, reduce production costs and increase manufacture yield [20]. To confirm that our assay can identify an increase in viral fusion, we included TMPRSS2 in the transfection mix with ACE2 (Fos-ACE2-TMPRSS2). As expected, adding this serine protease increased the observed fluorescent signal (Fig. 1B). These results confirm that our setup could identify compounds that enhance viral-cellular membrane fusion.

Next, we adapted our methodology for the HTS of small molecules and verified its suitability by the Z′ factor (0.63 or 0.62 for the Jun-S + Fos-ACE2 or Jun-ACE2 + Fos-S combinations, respectively) [21]. Briefly, Hek 293T cells were transfected with Jun-S or Fos-ACE2 plus a plasmid encoding the Renilla luciferase under a constitutive promoter. After transfection, cell pools were mixed as described above, seeded at 96 well plates, and treated with the appropriate small molecule. Each plate included a column of wells treated with DMSO. Additionally, plates included 8 wells as a negative control containing cells transfected with Jun or Fos (both cell pools were combined as previously) and treated with DMSO. Once treated, cells were incubated for 48 h, followed by fluorescence measurement and hit selection (Fig. 2A).

**Fig. 3:**
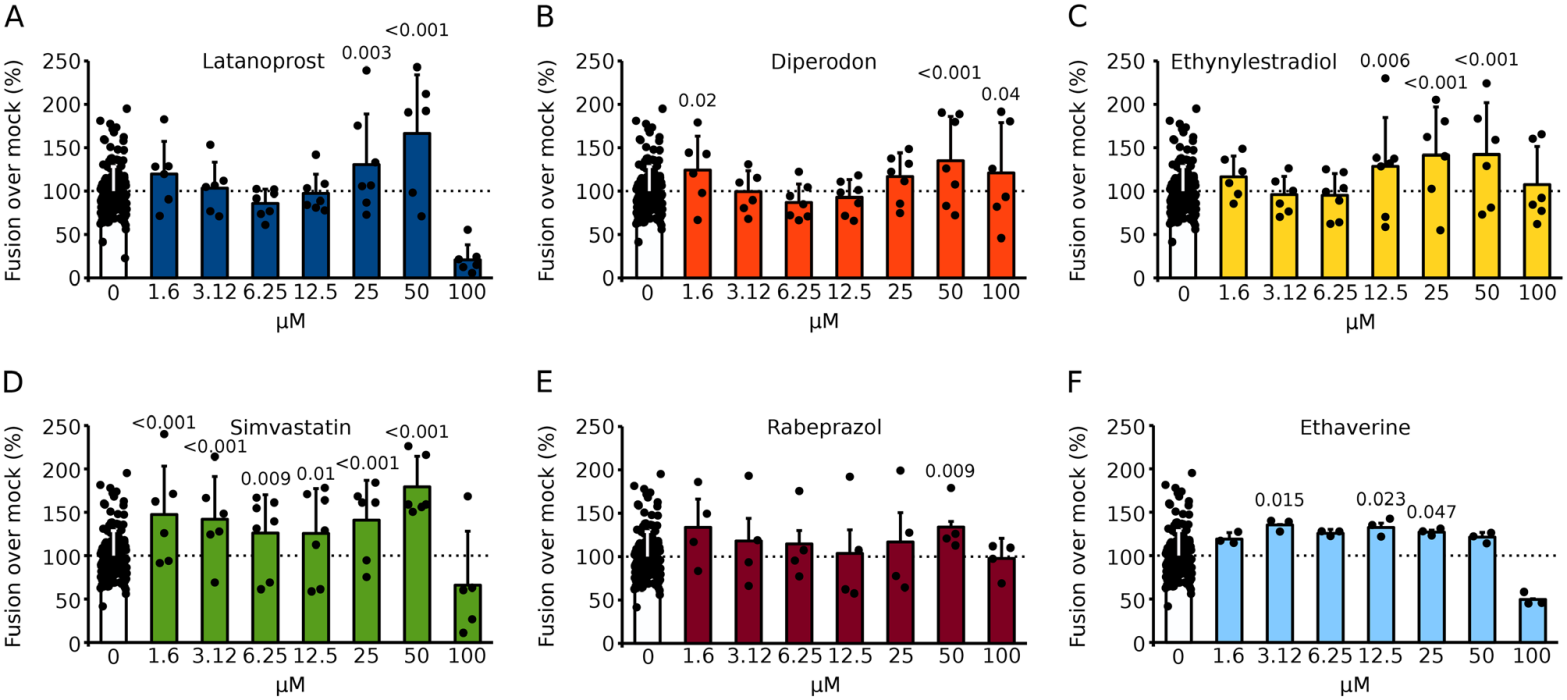
Analysis of hit compounds. **A–F** Selected compounds were repurchased, and their ability to increase syncytia formation was tested with the BiMuC assay. Briefly, Hek293T cells were transfected with Jun plus SARS-CoV 2 S proteins and independently with Fos and human ACE2. After 24 h, samples were combined and treated with the appropriate compound at the indicated concentration or mock treated for 48 h. Next, the fluorescence, indicative of the percentage of fusion was calculated. The fluorescence percentages over the mock (DMSO) treated samples are represented. The average and standard deviation of at least three independent experiments are shown. Dots represent individual experiments. Based on a one-tailed homoscedastic t-test, statistical differences between compound and mock-treated samples were calculated. The p-value is shown when significant differences are found

A library consisting of 1280 chemically diverse small molecules (Prestwick Chemical, GreenPharma) was screened, and the results were standardized by the *Z-Score;* 76 primary hits were selected based on a *Z-Score* > 1.5 (Table S1). Hits were re-tested using the same experimental conditions and selection criteria. Only the compounds capable of enhancing viral fusion in both rounds were considered (Fig. 2B). Additionally, we measured the luminescence from the Renilla luciferase and eliminated those compounds promoting cell division or survival, thus increasing the fluorescence signal without effectively increasing cell-cell fusion. We also tested the fluorescence properties of the remaining hits and discarded those compounds emitting near 530 nm (Fig. 2B).

**Fig. 4:**
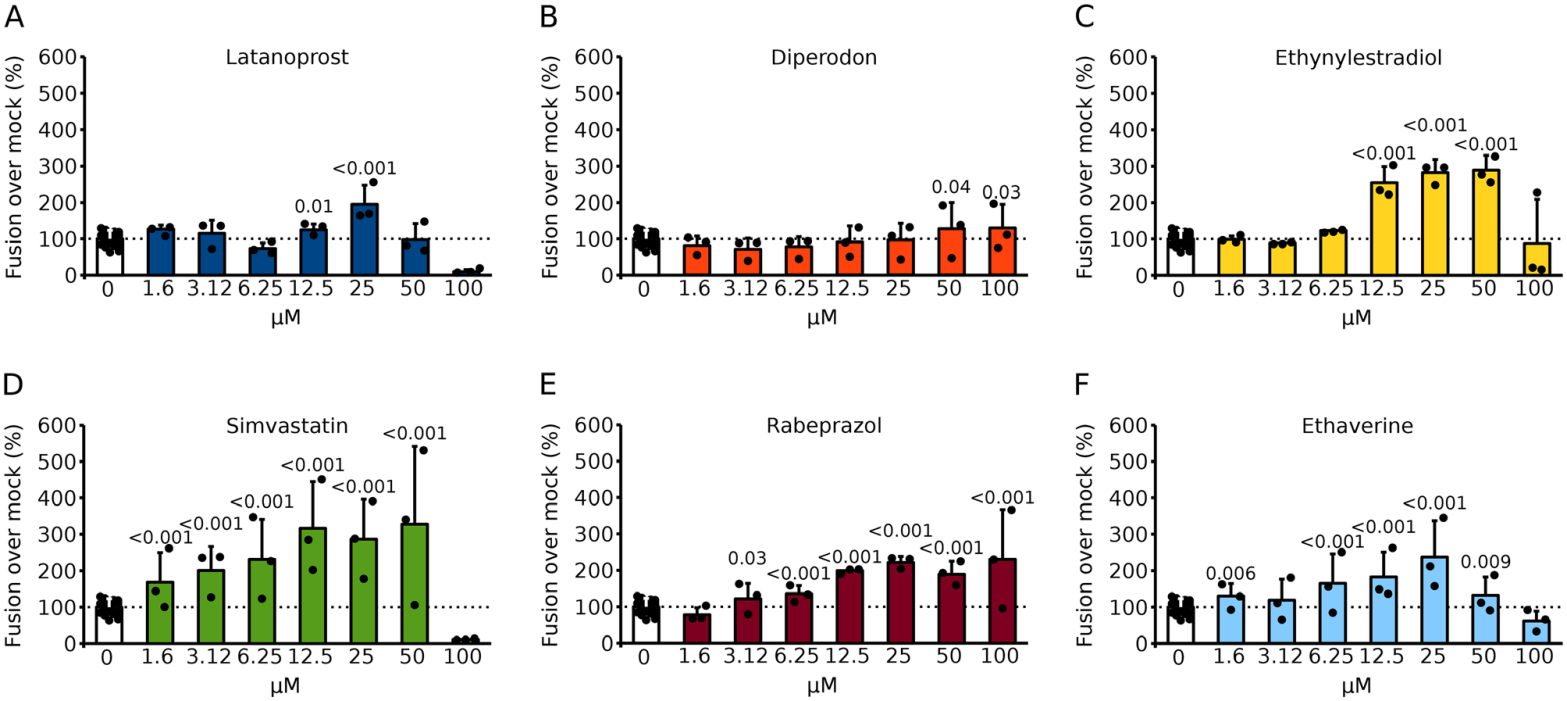
Analysis of hit compounds after 72 h of incubation. **A–F** The ability of the selected compounds to increase syncytia formation was tested with the BiMuC assay in Hek293T cells. Briefly, Hek293T cells were transfected with Jun plus SARS-CoV 2 S proteins and independently with Fos and human ACE2. After 24 h, samples were combined and treated with the appropriate compound at the indicated concentration or mock-treated for 72 h. The fluorescence percentages over the mock (DMSO) treated samples are represented. The average and standard deviation of at least three independent experiments are shown. Dots represent individual experiments. Based on a one-tailed homoscedastic t-test, statistical differences between compound and mock-treated samples were calculated. When differences are significant, the p-value is shown

A total of 6 compounds were selected; these were repurchased from a different vendor and re-tested at multiple concentrations to confirm their ability to enhance viral membrane fusion in Hek 293T. Compounds were incubated for 48 or 72 h (Figs. 3 and 4). We observed a modest increase in viral-induced membrane fusion when compounds were incubated for 48 h; latanoprost, simvastatin, and ethynylestradiol were the stronger inducers. However, after 72 h of incubation, most compounds (except diperodon) enhance the fluorescence signal. A limited dose response was observed when the compounds were incubated for 48 h. However, a stronger dose dependence was observed at 72 h post-treatment.

**Fig. 5:**
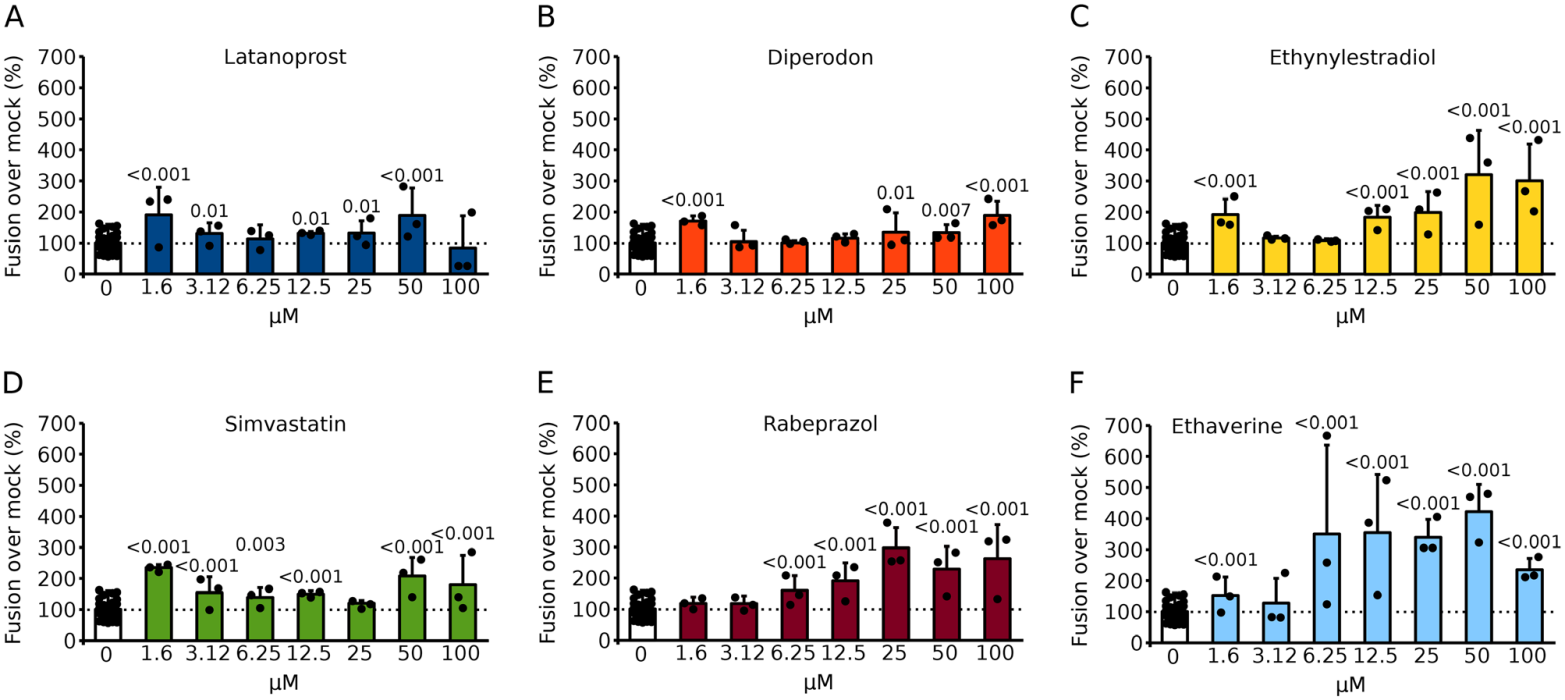
Analysis of hit compounds in the presence of TMPRSS2. **A–F** The ability of the selected compounds to increase syncytia formation in the presence of TMPRSS2 was tested with the BiMuC assay. Hek293T cells were transfected with TMPRSS2 alongside the viral and cellular machinery required for syncytia formation. Next, cells were mixed and treated with the appropriate compound for 48 h. The fluorescence percentages over the mock (DMSO) treated samples are represented. The average and standard deviation of at least three independent experiments are shown. Dots represent individual experiments. Based on a one-tailed homoscedastic t-test, statistical differences between compound and mock-treated samples were calculated. When differences are significant, the p-value is shown

Next, to identify whether the selected compounds could induce an increase in viral fusion under optimal growing conditions, we tested them in the presence of TMPRSS2 after 48 h of incubation. In this case, the enhancing effect of the compounds was substantially stronger than without the protease (Fig. 5). Of note, once the protease was added, a concentration-dependent response was observed.

**Fig. 6:**
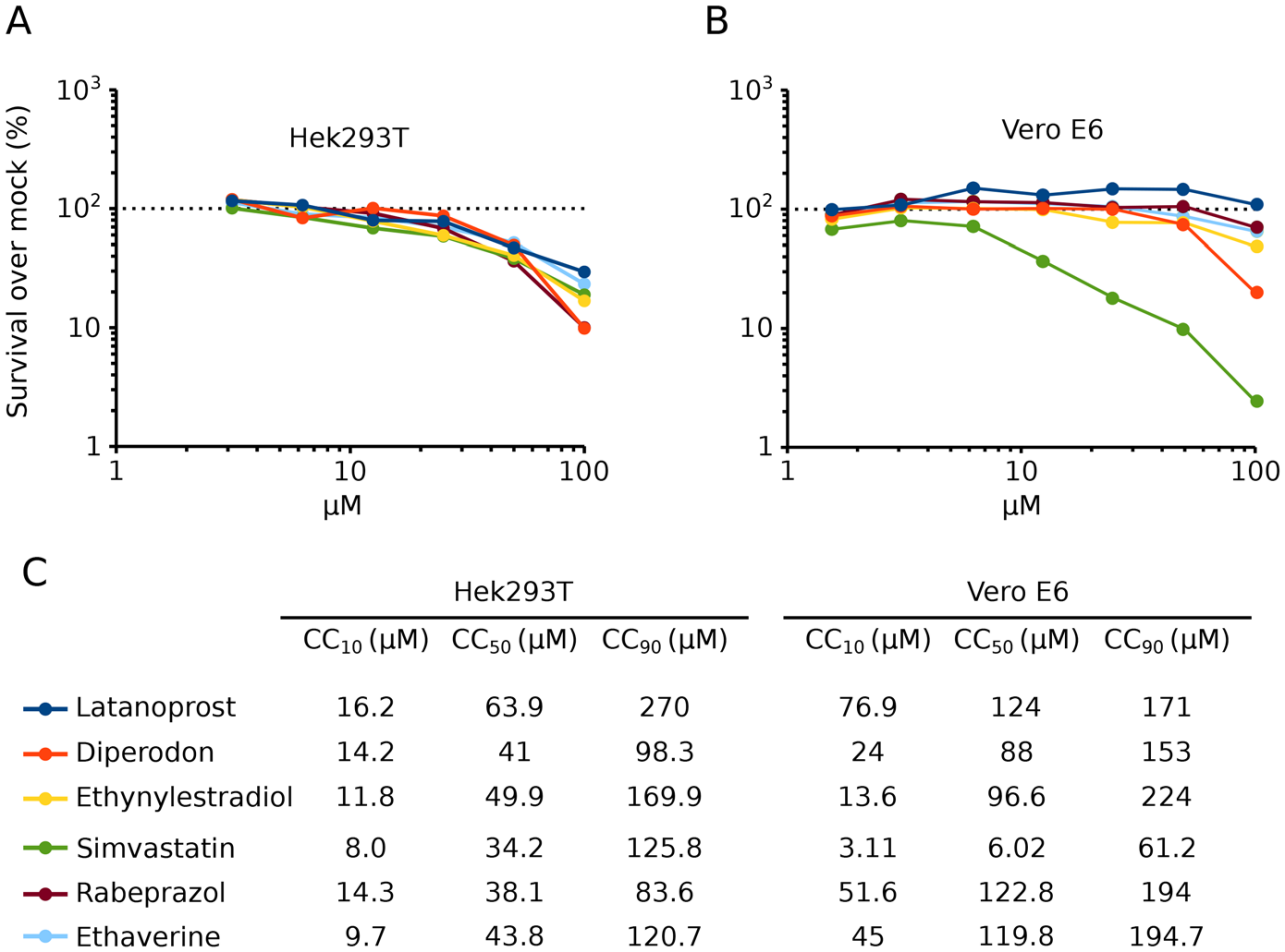
Toxicity of hit compounds. **A, B** The toxicity in Hek 293T and Vero E6 cells (**A** and **B**, respectively) was analyzed after incubating the compounds at the indicated concentration for 48 h. Toxicity was analyzed by measuring ATP levels using the Cell-Titer Glo assay (Promega) following the manufacturer’s indications. Dots represent the average of at least three independent experiments. **C** The CC10, CC50, and CC90 values were calculated with the data shown in panels A and B

We also assayed the toxicity of these 6 compounds by measuring the ATP content in the cells (Fig. 6). In Hek 293T cells, all six compounds demonstrated some toxicity at high concentrations. Interestingly, despite the toxicity at these concentrations, we observed an increase in cell-cell fusion. The CC_50_ and the cell-cell fusion activity at this concentration are shown in Table 1; the left column highlights an activity index (the cell-cell fusion percentage divided by the CC_50_). Additionally, the toxicity of ethynylestradiol was tested in the presence of TMPRSS2 (Fig. S2). The results indicate that the compound toxicity was not affected by the presence of the cellular protease. We also analyzed the toxicity of these compounds in Vero cells, a cell line widely utilized to grow viruses in vitro for vaccine purposes [15] (Fig. 6). In this cell line, simvastatin (CC_50_ ~ 6 µM) clearly showed toxicity above the rest of the compounds. Based on these results (effectiveness and toxicity) we decided to focus on ethaverine, rabeprazole, and ethynylestradiol from this point on.

**Table 1:** Activity index of selected compounds

		Fusion % at CC_50_		Activity index (Fusion % at CC_50_/CC_50_)	
	**CC**_**50**_ (µM)	**NO TMPRSS2**	**TMPRSS2**	**NO TMPRSS2**	**TMPRSS2**
**Latanoprost**	63,86	70,98	152,75	1,11	2,39
**Diperodon**	40,96	123,98	138,77	3,03	3,39
**Ethynylestradiol**	49,89	162,00	312,60	3,25	6,27
**Simvastatin**	34,17	172,00	163,57	5,03	4,79
**Rabeprazol**	38,08	179,16	243,51	4,70	6,39
**Ethaverine**	43,84	125,39	419,22	2,86	9,56

### Effect of ethaverine, rabeprazole, and ethynylestradiol on NiV-induced membrane fusion

**Fig. 7:**
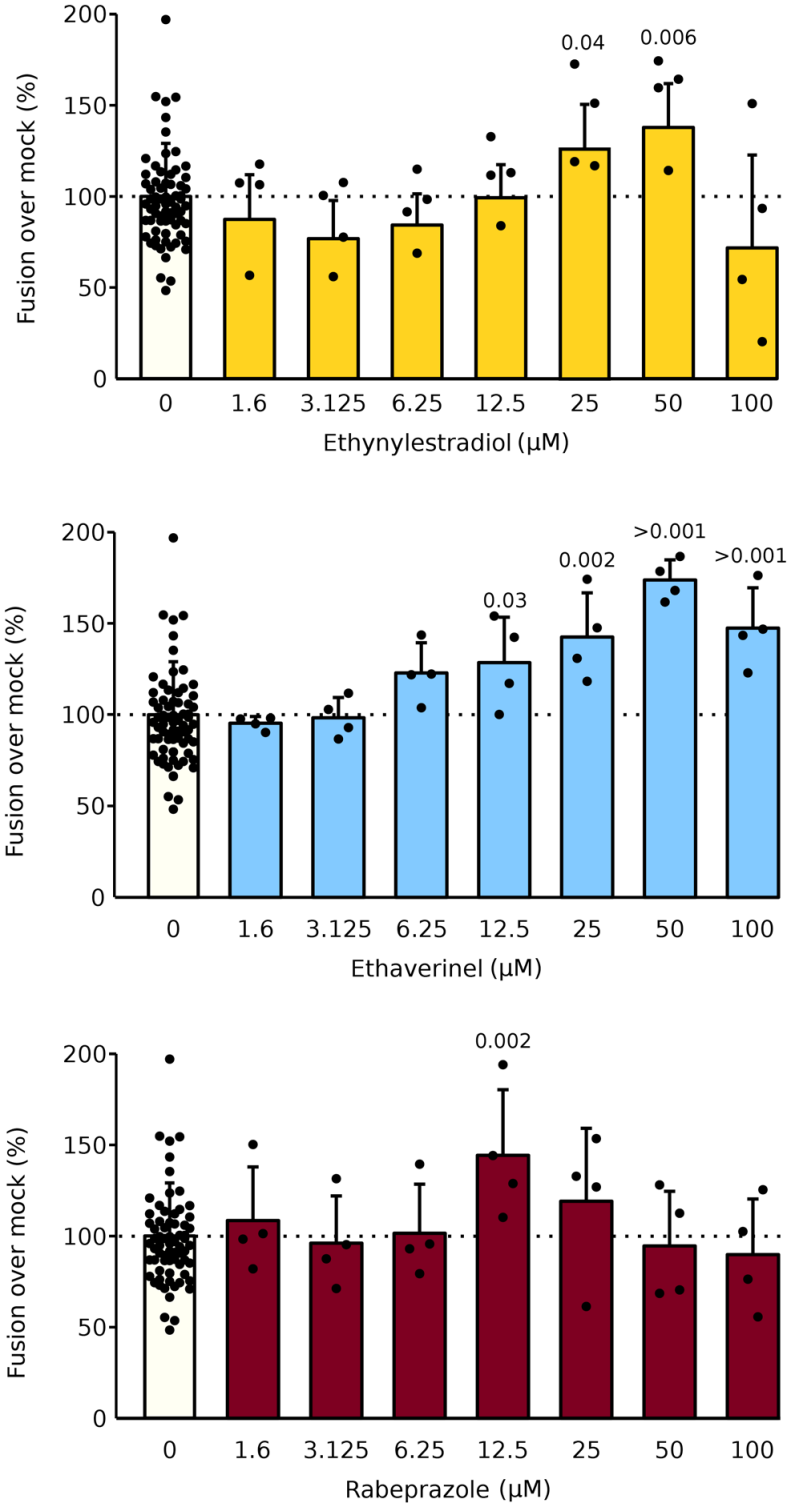
Effect of ethynylestradiol, ethaverine, and rabeprazole on Nipah virus membrane fusion. To test the effect of ethynylestradiol, ethaverine, and rabeprazole on NiV membrane fusion Hek 293T cells were transfected with plasmids encoding NiV F, G, and Jun or with Fos. Approximately 24 h later, cell pools were mixed, and the appropriate compound was added at the indicated concentrations. After 48 h, fluorescence was measured. Bars show the average and standard deviation of at least three independent experiments. Data are presented as fold over the mock-treated samples. Fos). Based on a one-tailed homoscedastic t-test (p-values are indicated above the corresponding bar, ns non-significant), statistical differences between compound and mock-treated samples were calculated. When differences were significant, the p-value was indicated

To further analyze the role of the selected compounds (ethynylestradiol, ethaverine, and rabeprazole) on the entry of enveloped viruses, we turned our attention to the Nipah virus (NiV). NiV, a member of the Paramyxoviridae family, requires two proteins to carry out the fusion of the viral and cellular membranes, the glycoprotein (G) that attaches to a receptor protein on the surface of the receptor cell and the fusion (F) protein which in turn will force the fusion of the cellular and viral membranes. NiV is a BSL4 restricted agent. Therefore, to test the effect of ethynylestradiol, ethaverine, and rabeprazole we utilized the BiMuC assay adapted to the study of NiV entry [8]. Our results indicate, once again, that all three of these compounds can enhance viral-mediated membrane fusion in a mild dose-dependent manner (Fig. 7).

### Effect of ethaverine, rabeprazole, and ethynylestradiol on the growth of SARS-CoV-2 and influenza A virus

**Fig. 8:**
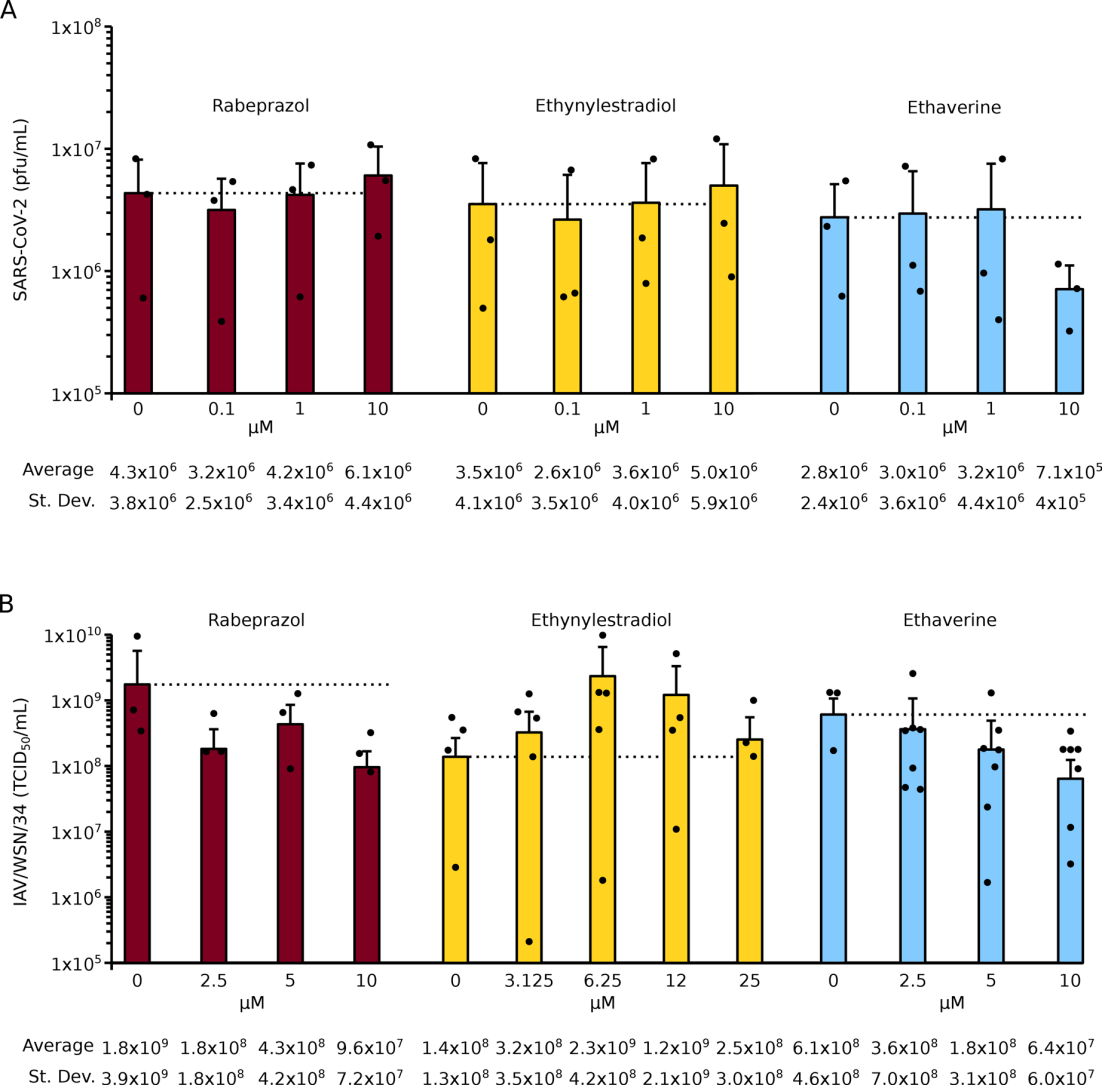
Effect of selected compounds on SARS-CoV-2 and IAV. **A** SARS-CoV-2 was grown at a multiplicity of infection (MOI) of 0.01 in Vero E6 cells for 48 h in the absence or presence of ethaverine, rabeprazole, or ethynylestradiol at the indicated concentrations. Next, supernatants were collected and tittered by standard plaque assay in Vero E6 cells. Bars show the average plaque forming units (pfu) and standard deviation of at least three independent experiments. Dots represent individual experiments. No statistical differences, based on a two-tailed homoscedastic t-test, were found between treated and mock-treated samples. **B** IAV/WSN/33, at an MOI of 0.001, was used to infect Madin-Darby canine kidney (MDCK) cells. IAV was incubated in the absence or presence of ethaverine, rabeprazole, or ethynylestradiol at the indicated concentrations for 48 h. Next, supernatants were collected and tittered by the 50% Tissue Culture Infective Dose (TCID_50_) method in MDCK cells. Bars show the average plaque forming units (pfu) and standard deviation of at least three independent experiments. Dots represent individual experiments. Based on a two-tailed homoscedastic t-test, no statistical differences were found between treated and mock-treated samples

Our assay is based on syncytia formation, which might not accurately recapitulate the fusion of the viral and cellular membranes during an infection. Consequently, at this point, we decide to analyze the effect of the hit compounds on the viral growth of SARS-CoV-2 [18, 22, 23]. Our results indicated that rabeprazole and ethynylestradiol increased viral titers at 48 h post-infection at concentrations below the CC_50_ (Fig. 8A) although no significant differences were found between treated and untreated samples. On the other hand, ethaverine did not increase or accelerate the growth of SARS-CoV-2 (Fig. 8A).

Next, we analyzed if the ability of these compounds to enhance viral growth was restricted to SARS-CoV-2. To do so, we tested the effect of ethaverine, rabeprazole, and ethynylestradiol on the growth of the Influenza A virus (IAV). IAV causes substantial annual morbidity and mortality with seasonal epidemic outbreaks. Seasonal influenza epidemics may be managed by vaccination, and multi-valent vaccines containing IAV and influenza B virus components are widely distributed annually. Like SARS-CoV-2, IAV is an enveloped virus that relies on a class I fusion protein, the hemagglutinin (HA) protein, for the fusion of the viral and cellular membranes.

IAV infections we carried out in Madin-Darby canine kidney (MDCK) cells in the presence and absence of the selected compounds [24]. Our results indicate that ethynylestradiol increases IAV titers by approximately one log (Fig. 8B), although no significant differences were found. On the other hand, ethaverine and rabeprazole did not increase viral titers (Fig. 8B).

Most of the compounds included in the chemical library used in our screen are approved drugs and are currently used in several treatments. However, it is important to note that our experiments are insufficient to claim that the hit compounds, including ethynylestradiol, might worsen the outcome of a patient during infection with an enveloped virus.

In summary, our results suggest that ethynylestradiol could be used in vitro to enhance the growth of enveloped viruses. Based on previous works [25–29], ethynylestradiol might alter the properties of the viral and cellular membranes to facilitate membrane fusion and or viral endocytosis. Compounds such as polybrene are used to improve retrovirus infections by facilitating the adhesion of virions to the cell surface since the addition of positively charged polycations reduces the repulsive forces between the cell and the virus, improving transduction efficiency [30]. However, as far as we are aware, no compounds that improve membrane fusion and subsequently increase and accelerate viral growth have been identified previously.

Our results demonstrate that BiMuC can be implemented to monitor the SARS-CoV-2 membrane fusion process. Furthermore, we show that this methodology is suitable for the high-throughput identification of small molecules that influence the viral entry of some enveloped viruses and, thus, viral growth.

## Electronic supplementary material

Below is the link to the electronic supplementary material.

Supplementary Material 1

Supplementary Material 2

**Figure S1**. SARS-CoV-2 S protein-induced syncytia. Hek 293T cells were transfected with SARS-CoV-2 S protein and human ACE2 coding plasmids (**A-C**) or mock transfected (**D**). After 24 hours cells were visualized using a Motic AE200 microscope with a 10x objective

**Figure S2.** Ethynylestradiol toxicity in the presence of TMPRRS2. **A and B**. To analyze the effect of TMPRSS2 on ethynylestradiol toxicity Hek 293T cells were transfected with TMPRR22 or eYFP and 48 hours later the toxicity was assayed. Toxicity was analyzed by measuring ATP levels using the Cell-Titer Glo assay (Promega) following the manufacturer’s indications. Dots represent the average of two independent experiments. The CC10, CC50, and CC90 values are indicated

## Data Availability

All study data are included in the article and/or Supplementary Information.

## References

[1] Wu F, Zhao S, Yu B, Chen Y-M, Wang W, Song Z-G (2020). A new coronavirus associated with human respiratory disease in China. Nature.

[2] Zhou P, Yang X-L, Wang X-G, Hu B, Zhang L, Zhang W (2020). A pneumonia outbreak associated with a new coronavirus of probable bat origin. Nature.

[3] Kim D, Lee J-Y, Yang J-S, Kim JW, Kim VN, Chang H (2020). The Architecture of SARS-CoV-2 Transcriptome. Cell.

[4] Wrapp D, Wang N, Corbett KS, Goldsmith JA, Hsieh C-L, Abiona O (2020). Cryo-EM structure of the 2019-nCoV spike in the prefusion conformation. Science.

[5] Yang H, Rao Z (2021). Structural biology of SARS-CoV-2 and implications for therapeutic development. Nat Rev Microbiol.

[6] Wang Q, Zhang Y, Wu L, Niu S, Song C, Zhang Z (2020). Structural and functional basis of SARS-CoV-2 entry by using human ACE2. Cell.

[7] Rando HM, Lordan R, Lee AJ, Naik A, Wellhausen N, Sell E, et al. Application of traditional Vaccine Development Strategies to SARS-CoV-2. mSystems. Volume 0. American Society for Microbiology; 2023. pp. e00927–22. 10.1128/msystems.00927-22 PMC10134813 36861991

[8] García-Murria MJ, Expósito-Domínguez N, Duart G, Mingarro I, Martinez-Gil L. A Bimolecular Multicellular Complementation System for the detection of Syncytium formation: a New Methodology for the identification of Nipah Virus Entry inhibitors. Viruses. 2019;11. 10.3390/v11030229 PMC6466393 30866435

[9] Islam MA, Park T-E, Singh B, Maharjan S, Firdous J, Cho M-H (2014). Major degradable polycations as carriers for DNA and siRNA. J Control Release Off J Control Release Soc.

[10] Grau B, Javanainen M, García-Murria MJ, Kulig W, Vattulainen I, Mingarro I (2017). The role of hydrophobic matching on transmembrane helix packing in cells. Cell Stress.

[11] Kerppola TK (2006). Design and implementation of bimolecular fluorescence complementation (BiFC) assays for the visualization of protein interactions in living cells. Nat Protoc.

[12] Magliery TJ, Wilson CGM, Pan W, Mishler D, Ghosh I, Hamilton AD (2005). Detecting protein-protein interactions with a green fluorescent protein fragment reassembly trap: scope and mechanism. J Am Chem Soc.

[13] García-Murria MJ, Duart G, Grau B, Diaz-Beneitez E, Rodríguez D, Mingarro I (2020). Viral Bcl2s’ transmembrane domain interact with host Bcl2 proteins to control cellular apoptosis. Nat Commun.

[14] van Dam H, Castellazzi M (2001). Distinct roles of Jun: Fos and Jun : ATF dimers in oncogenesis. Oncogene.

[15] Barrett PN, Terpening SJ, Snow D, Cobb RR, Kistner O (2017). Vero cell technology for rapid development of inactivated whole virus vaccines for emerging viral diseases. Expert Rev Vaccines.

[16] Kiesslich S, Kamen AA (2020). Vero cell upstream bioprocess development for the production of viral vectors and vaccines. Biotechnol Adv.

[17] Tapia F, Vázquez-Ramírez D, Genzel Y, Reichl U (2016). Bioreactors for high cell density and continuous multi-stage cultivations: options for process intensification in cell culture-based viral vaccine production. Appl Microbiol Biotechnol.

[18] Matsuyama S, Nao N, Shirato K, Kawase M, Saito S, Takayama I (2020). Enhanced isolation of SARS-CoV-2 by TMPRSS2-expressing cells. Proc Natl Acad Sci U S A.

[19] Hoffmann M, Kleine-Weber H, Schroeder S, Krüger N, Herrler T, Erichsen S (2020). SARS-CoV-2 cell entry depends on ACE2 and TMPRSS2 and is blocked by a clinically proven protease inhibitor. Cell.

[20] Fang Z, Lyu J, Li J, Li C, Zhang Y, Guo Y et al. Application of bioreactor technology for cell culture-based viral vaccine production: Present status and future prospects. Front Bioeng Biotechnol [Internet]. 2022 [cited 2022 Sep 20];10. Available from: https://www.frontiersin.org/articles/10.3389/fbioe.2022.921755. 10.3389/fbioe.2022.921755 PMC9395942 36017347

[21] Zhang J, Chung T, Oldenburg K (1999). A simple statistical parameter for use in evaluation and validation of high throughput screening assays. J Biomol Screen.

[22] Case JB, Bailey AL, Kim AS, Chen RE, Diamond MS (2020). Growth, detection, quantification, and inactivation of SARS-CoV-2. Virology.

[23] Zaliani A, Vangeel L, Reinshagen J, Iaconis D, Kuzikov M, Keminer O (2022). Cytopathic SARS-CoV-2 screening on VERO-E6 cells in a large-scale repurposing effort. Sci Data Nature Publishing Group.

[24] Martinez-Gil L, Alamares-Sapuay JG, Ramana Reddy MV, Goff PH, Premkumar Reddy E, Palese P (2013). A small molecule multi-kinase inhibitor reduces influenza a virus replication by restricting viral RNA synthesis. Antiviral Res.

[25] Bossard R, Stieger B, O’Neill B, Fricker G, Meier PJ (1993). Ethinylestradiol treatment induces multiple canalicular membrane transport alterations in rat liver. J Clin Invest.

[26] Cuevas MJ, Mauriz JL, Almar M, Collado PS, González-Gallego J (2001). Effect of epomediol on ethinyloestradiol-induced changes in bile acid and cholesterol metabolism in rats. Clin Exp Pharmacol Physiol.

[27] Elias E, Iqbal S, Knutton S, Hickey A, Coleman R (1983). Increased tight junction permeability: a possible mechanism of oestrogen cholestasis. Eur J Clin Invest.

[28] Miccio M, Orzes N, Lunazzi GC, Gazzin B, Corsi R, Tiribelli C (1989). Reversal of ethinylestradiol-induced cholestasis by epomediol in rat. The role of liver plasma-membrane fluidity. Biochem Pharmacol.

[29] Van Dyke RW, Root KV (1993). Ethinyl estradiol decreases acidification of rat liver endocytic vesicles. Hepatol Baltim Md.

[30] Cornetta K, Anderson WF (1989). Protamine sulfate as an effective alternative to polybrene in retroviral-mediated gene-transfer: implications for human gene therapy. J Virol Methods.

