# Supplementary material for: Identification of small molecules capable of enhancing viral membrane fusion
Source: Virol J. 2023 May 24;20:99. doi: 10.1186/s12985-023-02068-1 (PMC10206591; doi:10.1186/s12985-023-02068-1)
Supplement: Supplementary file 1 — Supplementary Material 1 [file 12985_2023_2068_MOESM1_ESM.docx]

**Table S1. Primary screen hits**

| **Compound** | **Z-score** |  | **Compound** | **Z-score** |  | **Compound** | **Z-score** |
| --- | --- | --- | --- | --- | --- | --- | --- |
| 5-fluorouracil | 3,16 |  | Doxycycline hydrochloride | 3,00 |  | Mometasone furoate | 2,51 |
| Acetopromazine maleate salt | 1,71 |  | Enilconazole | 3,25 |  | Mupirocin | 1,57 |
| Albendazole | 1,67 |  | Equilin | 1,98 |  | Nitrofurantoin | 2,23 |
| Altrenogest | 2,61 |  | Estramustine | 3,30 |  | Nocodazole | 2,99 |
| Antimycin A | 4,08 |  | Ethaverine hydrochloride | 4,10 |  | Oxfendazol | 1,63 |
| Atorvastatin | 1,88 |  | Ethynylestradiol 3-methyl ether | 1,52 |  | Oxibendazol | 2,13 |
| Azaguanine-8 | 6,79 |  | Floxuridine | 4,71 |  | Oxytetracycline dihydrate | 2,06 |
| Azapropazone | 1,91 |  | Flubendazol | 3,95 |  | Papaverine hydrochloride | 4,33 |
| Azelastine hydrochloride | 1,52 |  | Fusidic acid sodium salt | 2,35 |  | Parbendazole | 2,56 |
| Besifloxacin hydrochloride | 2,15 |  | Gefitinib | 3,33 |  | Pentamidine isethionate | 6,07 |
| Bromocryptine mesylate | 5,89 |  | Hexachlorophene | 3,99 |  | Pentetic acid | 2,14 |
| Carmofur | 4,69 |  | Hexestrol | 2,65 |  | Perhexiline maleate | 2,68 |
| Chloramphenicol | 2,64 |  | Homochlorcyclizine dihydrochloride | 2,19 |  | Practolol | 1,57 |
| Cladribine | 1,60 |  | Ibudilast | 1,71 |  | Rabeprazole Sodium salt | 3,89 |
| Clofilium tosylate | 2,76 |  | Imatinib | 2,20 |  | Sarafloxacin | 2,16 |
| Colchicine | 1,93 |  | Ipriflavone | 2,37 |  | Simvastatin | 1,72 |
| Crotamiton | 2,94 |  | Irinotecan hydrochloride trihydrate | 4,23 |  | Sparfloxacin | 2,09 |
| Cyclosporin A | 3,37 |  | Lansoprazole | 3,43 |  | Thiamphenicol | 2,42 |
| Dacarbazine | 2,34 |  | Maprotiline hydrochloride | 2,05 |  | Thioproperazine dimesylate | 2,39 |
| Daunorubicin hydrochloride | 1,79 |  | Meclocycline sulfosalicylate | 5,55 |  | Thiostrepton | 5,52 |
| Dequalinium dichloride | 4,90 |  | Merbromin | 8,74 |  | Tiratricol, 3,3',5-triiodothyroacetic acid | 4,40 |
| Desloratadine | 1,59 |  | Methiazole | 3,16 |  | Tosufloxacin hydrochloride | 1,77 |
| Desonide | 1,59 |  | Mevastatin | 1,92 |  | Tyloxapol | 3,89 |
| Docetaxel | 1,72 |  | Minocycline hydrochloride | 4,43 |  | Vorinostat | 4,62 |
| Doxorubicin hydrochloride | 4,48 |  | Mizolastine | 2,15 |  | Zaprinast | 1,64 |
|  |  |  |  |  |  | Zoledronic acid hydrate | 2,27 |
