# Supplementary material for: Identification of small molecules capable of enhancing viral membrane fusion
Source: Virol J. 2023 May 24;20:99. doi: 10.1186/s12985-023-02068-1 (PMC10206591; doi:10.1186/s12985-023-02068-1)
Supplement: Supplementary file 2 — Supplementary Material 2 [file 12985_2023_2068_MOESM2_ESM.pdf]

Table S1. Primary screen hits

Compound	Z-score
5-fluorouracil	3,16
Acetopromazine maleate salt	1,71
Albendazole	1,67
Altrenogest	2,61
Antimycin A	4,08
Atorvastatin	1,88
Azaguanine-8	6,79
Azapropazone	1,91
Azelastine hydrochloride	1,52
Besifloxacin hydrochloride	2,15
Bromocryptine mesylate	5,89
Carmofur	4,69
Chloramphenicol	2,64
Cladribine	1,60
Clofilium tosylate	2,76
Colchicine	1,93
Crotamiton	2,94
Cyclosporin A	3,37
Dacarbazine	2,34
Daunorubicin hydrochloride	1,79
Dequalinium dichloride	4,90
Desloratadine	1,59
Desonide	1,59
Docetaxel	1,72
Doxorubicin hydrochloride	4,48

Compound	Z-score
Doxycycline hydrochloride	3,00
Enilconazole	3,25
Equilin	1,98
Estramustine	3,30
Ethaverine hydrochloride	4,10
Ethynylestradiol 3-methyl ether	1,52
Floxuridine	4,71
Flubendazol	3,95
Fusidic acid sodium salt	2,35
Gefitinib	3,33
Hexachlorophene	3,99
Hexestrol	2,65
Homochlorcyclizine dihydrochloride	2,19
Ibudilast	1,71
Imatinib	2,20
Ipriflavone	2,37
Irinotecan hydrochloride trihydrate	4,23
Lansoprazole	3,43
Maprotiline hydrochloride	2,05
Meclocycline sulfosalicylate	5,55
Merbromin	8,74
Methiazole	3,16
Mevastatin	1,92
Minocycline hydrochloride	4,43
Mizolastine	2,15

Compound	Z-score
Mometasone furoate	2,51
Mupirocin	1,57
Nitrofurantoin	2,23
Nocodazole	2,99
Oxfendazol	1,63
Oxibendazol	2,13
Oxytetracycline dihydrate	2,06
Papaverine hydrochloride	4,33
Parbendazole	2,56
Pentamidine isethionate	6,07
Pentetic acid	2,14
Perhexiline maleate	2,68
Practolol	1,57
Rabeprazole Sodium salt	3,89
Sarafloxacin	2,16
Simvastatin	1,72
Sparfloxacin	2,09
Thiamphenicol	2,42
Thioproperazine dimesylate	2,39
Thiostrepton	5,52
Tiratricol, 3,3',5-triiodothyroaceti c acid	4,40
Tosufloxacin hydrochloride	1,77
Tyloxapol	3,89
Vorinostat	4,62
Zaprinast	1,64
Zoledronic acid hydrate	2,27
